# Alcohol-based surgical hand preparation: translating scientific evidence into clinical practice

**DOI:** 10.1186/s13756-018-0372-7

**Published:** 2018-07-09

**Authors:** Gilberto G. Gaspar, Mayra G. Menegueti, Ana Elisa R. Lopes, Roberto O. C. Santos, Thamiris R. de Araújo, Aline Nassiff, Lécio R. Ferreira, Maria Eulalia L. V. Dallora, Silvia R. M. S. Canini, Fernando Bellissimo-Rodrigues

**Affiliations:** 1 Infection Control Service, University Hospital of the Ribeirão Preto Medical School, University of São Paulo, Ribeirão Preto, SP Brazil; 2 Department of Surgery and Anatomy, Ribeirão Preto Medical School, University of São Paulo, Ribeirão Preto, SP Brazil; 3 Department of Fundamental Nursing, Ribeirão Preto College of Nursing, University of São Paulo, Ribeirão Preto, SP Brazil; 4 Hospital Administration, University Hospital of the Ribeirão Preto Medical School, University of São Paulo, Ribeirão Preto, SP Brazil; 5 Social Medicine Department, Ribeirão Preto Medical School, University of São Paulo, Ribeirão Preto, SP Brazil; 6 University Hospital of Ribeirão Preto Medical School, Avenida Bandeirantes, 3900 – Vila Monte Alegre, Ribeirão Preto, SP 14048-900 Brazil

## Abstract

**Background:**

Although alcohol-based surgical hand preparation offers potential advantages over the traditional surgical scrubbing technique, implementing it may be challenging due to resistance of surgeons in changing their practice. We aimed to implement alcohol-based surgical hand preparation in the hospital setting evaluating the impact of that on the quality and duration of the procedure, as well as on the prevention of surgical site infections.

**Methods:**

A quasi-experimental study conducted at a tertiary-care university hospital from April 01 to November 01, 2017. Participants were cardiac and orthopedic surgical teams (*n* = 56) and patients operated by them (*n* = 231). Intervention consisted of making alcohol-based handrub available in the operating room, convincing and training surgical teams for using it, promoting direct observation of surgical hand preparation, and providing aggregated feedback on the quality of the preparation. The primary study outcome was the quality of the surgical hand preparation, inferred by the compliance with each one of the steps predicted in the World Health Organization (WHO) technique, evaluated through direct observation. Secondary study outcome was the patient’s individual probability of developing surgical site infection in both study periods. We used the Wilcoxon for paired samples and McNemar’s test to assess the primary study outcome and we build a logistic regression model to assess the secondary outcome.

**Results:**

We observed 534 surgical hand preparation events. Among 33 participants with full data available for both study periods, we observed full compliance with all the steps predicted in the WHO technique in 0.03% (1/33) of them in the pre-intervention period and in 36.36% (12/33) of them in the intervention period (OR:12.0, 95% CI: 2. 4-59.2, *p* = 0.002). Compared to the pre-intervention period, the intervention reduced the duration of the preparation (4.8 min vs 2.7 min, respectively; *p* < 0.001). The individual risk of developing a surgical site infection did not significantly change between the pre-intervention and the intervention phase (Adjusted RR = 0.66; 95% CI 0. 16-2.70, *p* = 0.563).

**Conclusion:**

Our results demonstrate that, when compared to the traditional surgical scrub, alcohol-based surgical hand preparation improves the quality and reduces the duration of the preparation, being at least equally effective for the prevention of surgical site infections.

## Background

Surgical site infection (SSI) is a worldwide concern. A study conducted in 16 European countries identified that 20% of all notified healthcare-associated infections were related to surgical procedures [1]. In Florida, SSI represented one-third of all cases of healthcare-associated infections [2].

Surgical hand preparation is recommended by both the Centers for Disease Control and Prevention (CDC) and the World Health Organization (WHO) for preventing SSI in all kinds of surgical procedures [3]. There are two well-recognized methods for performing surgical hand preparation. The most traditional one is scrubbing hands and forearms with antimicrobial soap, usually 2% chlorhexidine or 10% povidone-iodine (PVPI). More recently, alcohol-based surgical hand preparation has been proposed as an alternative to surgical scrub [3–8]. Among potential advantages of the alcohol-based procedure are: less skin irritation, less time-consuming, economy of tap water, and more potent antimicrobial effect [9].

However, surgeons may be skeptical about adopting alcohol-based surgical hand preparation, and this may represent a challenge for implementing such a strategy in the operating room. A study carried out among 156 healthcare professionals in a medical center in Taiwan identified that a higher number of nurses employed alcohol-based handrub (ABHR) for surgical hand preparation as compared to surgeons. The authors attributed this finding to the greater familiarity of surgeons with the traditional surgical hand preparation technique and to their higher reliability on the antiseptic effect thereof [10].

Therefore, studies performed in the real world scenario are necessary to confirm the benefits of using ABHR for surgical hand preparation, predicted mostly by lab studies. Implementation research studies entail: (i) testing strategies to face obstacles and (ii) determining the best strategy to introduce innovations or to promote sustainable changes [11].

The present study aimed to implement the exchange of using antimicrobial soap for using ABHR in the context of surgical hand preparation and to evaluate the impact of that on the quality and duration of the procedure, as well as on the prevention of surgical site infections.

## Methods

This quasi-experimental study was conducted from April 01 to November 01, 2017 in a tertiary-care public-affiliated university hospital in Brazil. The Research Ethics Committee of the study institution approved its protocol before the study implementation (n° 64,964,217.9.0000.5440).

The study population consisted of all members of the cardiac and orthopedic surgical teams of the study facility and all patients operated by them during the study period. Each patient was included just one time in the study, so re-operations during the study period were not considered in the data analysis.

In the pre-intervention period, which lasted 3 months, traditional surgical scrub was performed with antimicrobial soap (either 2% chlorhexidine or 10% PVPI) before every surgery and ABHR was not available for surgical hand preparation. Direct observation of the procedures was implemented as a part of the data collection.

Just after the pre-intervention period, we started the implementation period, which lasted 1 month, and during it no data was collected. The intervention consisted of making ABHR available in the operating room, convincing and training the selected surgical teams for using it, promoting direct observation of surgical hand preparation, and providing them aggregated feedback on the quality of the preparation. The training consisted of a 4 h workshop, repeated five times, when the scientific literature about surgical hand preparation was reviewed and the participants were instructed about how to apply ABHR for this purpose, according to the WHO technique. To ensure the covering of all hand and forearms surface, we asked all participants to apply a fluorescent ABHR simulating the surgical hand preparation, which was afterwards revealed by a fluorescence apparatus.

Just after the implementation period, the intervention period began, and the data collection re-started. The intervention period also lasted 3 months.

The primary study outcome was the quality of the surgical hand preparation, inferred by the compliance with each one of the steps predicted in the WHO technique, evaluated through direct observation in the operating room. Secondary study outcome was the patient’s individual probability of developing SSI in both study periods.

The following instruments were employed for data collection:

1. Surgical site infection (SSI) – we followed up operated patients for 1 month to verify whether they had developed SSI or not, according to the CDC criteria, including post-discharge surveillance. Patients were screened by an infection control nurse, and SSI episodes were confirmed by an infectious disease specialist of the Infection Control Service.
2. Risk factors for SSI – an instrument containing the following items was designed: study phase, surgical procedure, extracorporeal circulation time (min), surgical time (min), American Society of Anesthesiologists physical status classification system (ASA), comorbidities, use of immunosuppressive drugs, Body Mass Index (BMI), preoperative hospital stay (in days), presence of infection before the surgery, antibiotic prophylaxis, surgical complications and SSI by a multidrugresistant microorganism (carbapenem-resistant Enterobacteria, *Pseudomonas* spp., or *Acinetobacter baumannii*; methicillin-resistant *Staphylococcus aureus*; or vancomycin-resistant *Enterococcus* spp.). Two investigators from the Infection Control Service collected these data.
3. Surgical hand-preparation quality assessment – an instrument based on the WHO technique and framing the following items was designed: time spent on surgical hand preparation, use of jewelry (e.g., rings, wristwatch, and bracelets), short nails, and compliance with all the antisepsis steps (palm to palm; right palm over back of left hand and vice-versa; interdigital spaces, thumb, nails, fingertips, wrist, and forearm). Two investigators with extensive training in hand hygiene and surgical hand preparation performed these observations in both study periods.

Taking into account the previously unknown baseline status of the quality of the surgical hand preparation (primary study outcome) in the study facility, we could not estimate a sample size for the study. The period of 3 months for the pre-intervention and 3 months for the intervention was chosen for convenience. Data were analyzed with the STATA SE® software version 14. First, a descriptive analysis was accomplished. Then, we calculated the weighted average for each surgical team member of the compliance with each one of the steps of the WHO technique for surgical hand preparation (primary study outcome) and we used the Wilcoxon test for paired samples to compare it between the two study periods. We also used McNemar’s test to compare full compliance with the WHO technique observed before and after the intervention. Finally, we build a logistic regression model to assess the secondary study outcome (SSI risk) on the patient level, adjusting it for surrogate markers of baseline severity status, such as pre-operative length of stay, ASA score, and other potential risk factors for SSI implicated in univariate analysis (*p* < 0.10).

## Results

All cardiac and orthopedic surgery staff members agreed to participate in the study and were included in the pre-intervention and intervention phases, totaling 56 participants. All the 231 patients who had undergone cardiac (85 patients) or orthopedic (146 patients) surgeries during the study period were also included.

Table 1 contains a descriptive analysis of selected demographical and clinical characteristics of the patients operated in pre-intervention (132 patients) and intervention (99 patients) phases. Although a reasonable balance was observed for most of these variables between the two study periods, we have detected some important differences. Median age was higher in the pre-intervention (53.7 years old) than in the intervention phase (46.9 years old). Orthopedic surgery was less frequent in the pre-intervention (57.6%) than in the intervention phase (70.7%). The average duration of the procedures was shorter in the pre-intervention (median time: 175 min) compared to the intervention phase (median time: 240 min). Technical complications during procedure were more frequent in the pre-intervention phase (3.8% vs 1%) than in the intervention phase. In addition, finally, post-discharge follow-up reached more patients in the pre-intervention (86.4%) than in the intervention phase (73.7%).

**Table 1:** Demographical and clinical aspects of the patients operated in the pre-intervention and intervention phases of the study

Patients characteristics	Pre-intervention phase		Intervention phase	
	Surgical scrub (*n* = 132)		Alcohol-based surgical hand preparation (*n* = 99)	
	Number	Percent	Number	Percent
Sex				
Male	74	56.0	62	62.6
Female	58	44.0	37	37.4
Age (years)^a^	53.7 (25.0–65.9)		46.9 (20. 4-63.5)	
Surgical specialty				
Orthopedics	76	57.6	70	70.7
Cardiac	56	42.4	29	29.3
Post-discharge follow-up	114	86.4	73	73.7
Duration of surgery (min)^a^	180 (122–263)		200 (130–255)	
Duration of extracorporeal circulation (min)^a^ (*n* = 41)	115 (80–140.5)		125 (95–155)	
Preoperative length of stay (days)^a^	2 (1-8)		2 (1-4)	
Body Mass Index – BMI	26.7 (23. 8-31.7)		27.2 (23. 2-30.5)	
Technical complication during procedure	5	3.8	1	1
Urgent procedure	8	6	8	8.1
Chronic hepatopathy	2	1.5	5	5
Malignancy	10	7.6	5	5
Diabetes Mellitus	32	24.2	27	27.3
Use of immunosuppressive drug	5	3.8	2	2
Smoking	31	23.5	20	20.2

^a^Median (interquartile range)

### Surgical hand preparation quality assessment

We directly observed 534 surgical hand preparation events. In the pre-intervention phase, 303 events were observed, and in most of them 2% chlorhexidine (*n* = 180) was employed for surgical scrub and, in the remaining events, 10% PVPI (*n* = 123) was used. In the intervention phase, we observed 231 events of alcohol-based surgical hand preparation (Table 2). Compliance with most of the steps predicted by the WHO technique did not significantly varied between the two study periods. However, average compliance with the scrubbing of the thumb in the pre-intervention period (86.2%) was lower than the rubbing of the thumb in the intervention period (96.9%), and that difference was borderline statistically significant (*p* = 0.052). Nail scrubbing was observed in only 33.7% of events when chlorhexidine was employed and in only 41.5% of the events when PVPI was used. Interestingly, hands rinsing at the end of surgical hand preparation was performed only in 72.3% of the events in which PVPI was used. Another problem detected in the scrubbing period was that only 13.5 and 18.0% of the participants did not get back to hands after scrubbing the forearms, while using chlorhexidine and PVPI, respectively.

**Table 2:** Percentage of weighted average compliance with each one of the steps of the WHO technique for surgical hand preparation according to the study phase and product employed

Compliance with each one of the steps of the WHO technique for surgical hand preparation	Pre-intervention phase (*n* = 303)		Intervention phase (n = 231)	*P*-Value^**c**^
	Surgical scrub with 2% chlorhexidine (%) (*n* = 180)^**a**^	Surgical scrub with 10% PVPI (%) (*n* = 123)^**b**^	Alcohol-based surgical hand preparation (%) (*n* = 231)^**b**^	
Removed jewelry	99.4	100	100	1.000
Short nails	95	100	97.8	0.341
Finger	96.6	95.9	98.2	0.762
Palm	100	100	99	0.157
Back of hand	96.6	97.4	98.2	0.946
Interdigital space	82.1	85.5	93	0.948
Thumb	85.7	87.2	96.9	0.052
Wrist	95.5	98.3	99.1	0.247
Did not get back to hands after donning forearms	13.5	18	NA	NA
Nail scrubbing	33.7	41.5	NA	NA
Hands rinsing	98.8	72.3	NA	NA
Time (min)	4.8	4.9	2.7	0.001

*NA* not applicable

^**a**^The number of surgical hand preparation events observed per participant ranged from 1 to 24

^**b**^The number of surgical hand preparation events observed per participant ranged from 1 to 16

^**c**^Comparison between the pre-intervention and intervention phases by the Wilcoxon for paired samples test

Considering a subset of 33 participants with full data available for both study periods, we observed a full compliance with all the steps predicted in the WHO technique for surgical hand preparation in 0.03% (1/33) of them in the pre-intervention period and in 36.36% (12/33) of them in the intervention period (OR:12.0; 95% CI: 2. 4-59.2; *p* = 0.002).

The median scrubbing time was 4.8 min when the surgical team used chlorhexidine, and 4.9 min when they used PVPI. This was significantly longer than the median 2.7 min spent on rubbing hands and forearms, in the intervention period (*p* = 0.001).

### Analysis of potential risk factors for surgical site infection

We analyzed all 231 patients operated during the study period. Among patients operated in the pre-intervention period, 8.3% (11/132) developed SSI, while during the intervention period, SSI rate was reduced to 4.0% (4/99), although those differences did not reach statistical significance (RR = 0.48, 95% CI 0. 16-1.48).

Table 3 exhibits the univariate analysis of the potential risk factors for SSI observed during the study implementation. According to that analysis, no variable was implicated as a predictor of SSI and the alcohol-based surgical hand preparation, implemented in the intervention period, was not considered to impact on the individual probability of developing SSI (RR = 0.48, 95% CI 0. 16-1.48, *p* = 0.281).

**Table 3:** Univariate statistical analysis of risk factors for surgical site infection (SSI) among 231 patients operated during the study period

Variable	Patients characteristics		With SSI (*n* = 15)	Without SSI (*n* = 216)	Relative risk (95% CI) or *p*-value
	Situation	Total *n* (%)			
Chronic Hepatopathy	Present	4 (1.7%)	1 (6.6%)	3 (1.3%)	4.05 (0.68–23.80)
	Absent	227 (98.3%)			
Malignance	Present	15 (6.5%)	1 (6.6%)	14 (6.4%)	1.02 (0.14–7.30)
	Absent	216 (93.5%)			
Diabetes Mellitus	Present	59 (25.5%)	2 (13.3%)	57 (26.3%)	0.44 (0.10–1.92)
	Absent	172 (74.4%)			
Use of immunosuppressive drugs	Present	7 (3%)	1 (6.6%)	6 (2.7%)	2.28 (0.34–15.04)
	Absent	224 (97%)			
Smoking	Present	53 (22.9%)	5 (33.3%)	48 (22.2%	1.67 (0.60–4.69)
	Absent	178 (77.1%)			
Urgent surgery	Present	16 (6.9%)	1 (6.6%)	15 (6.9%)	0.95 (0.13–6.84)
	Absent	215 (93,1%)			
Technical complications during surgery	Present	8 (3.4%)	1 (6.6%)	7 (3.3%)	1.99 (0.29–13.34)
	Absent	223 (96.6%)			
Alcohol-based surgical hand preparation	Present	99 (42.8%)	4 (26.6%)	95 (43.9%)	0.48 (0.16–1.48)
	Absent	132 (57.2%)			
Extracorporeal circulation time (min)^**a**^			130 (65–210)	117 (80–145)	*p* = 0.688
ASA score^**a**^			2 (2-2)	2 (2-2)	*P =* 0.932
Pre-operative length of stay (days)^**a**^			3 (1–7)	2 (1–6)	*p* = 0.356
Duration of surgery (min)^**a**^			195 (129–285)	190 (125–261)	*p* = 0.778
Body Mass Index – BMI^**a**^			28.5 (25.3–30.1)	26.6 (23.6–31.2)	*p* = 0.802

^**a**^Median (interquartile range)

Table 4 presents the multivariate analysis of the impact that alcohol-based surgical hand preparation could have over the individual probability of developing SSI, adjusted for pre-operative length of stay and ASA score. This analysis was performed for a subset of 150 patients, for whom all the data, including ASA score, was available. According to that, alcohol-based surgical hand preparation was not predictor nor protective against the individual risk of developing SSI (adjusted RR = 0.66, 95% CI 0. 16-2.70, *p* = 0.563).

**Table 4:** Multivariate statistical analysis (logistic regression) of risk factors for surgical site infections (SSI) among 150 patients operated in the study period

Variable	Odds ratio (95% CI)	*P*-value
Alcohol-based surgical hand preparation	0.66 (0.16–2.70)	0.563
ASA score	1.09 (0.41–2.90)	0.862
Pre-operative length of stay (days)	0.92 (0.75–1.15)	0.493

The incidence of SSI caused by multidrug-resistant microorganisms was 6.06% (8/132) in the pre-intervention period, dropping to 1.01% (1/99) in the intervention period (RR = 0.17, 95% CI 0. 21-1.31, *p* = 0.082).

### Difficulties faced during implementation of an ABHR for surgical hand preparation

Two surgeons, one of the orthopedic surgery staff and another of the cardiac surgery staff, at first, reported that they did not rely on the ABHR effectiveness. Therefore, we scheduled meetings to show them that ABHR was actually efficient for surgical hand preparation. The professionals received all the information via email or printed articles. After this approach, these two surgeons complied with the use of ABHR during the intervention phase.

## Discussion

Many advantages of using ABHR for surgical hand preparation have been proposed by scientific literature, mostly based on laboratory-based studies [12–16]. Among those advantages, we can highlight less skin irritation, less time-consuming, economy of tap water, and more potent antimicrobial effect. However, only a few studies have attempted to implement and evaluate alcohol-based surgical hand preparation in a real world scenario [17, 18]. The present study adds evidence on this topic, confirming some of the predicted benefits. The studied intervention consisted of making ABHR available in the operating room, training surgical teams for using it, promoting direct observation of the preparation, and providing them aggregated feedback on the quality of the preparation. That intervention was demonstrated effective for enhancing the quality of the preparation, and for shortening the time spent on the preparation. Regarding SSI prevention, the intervention was proven at least equivalent to the previous protocol, focused on the surgical scrub with antimicrobial soap.

An observational study of traditional surgical hand preparation by scrubbing with chlorhexidine solution detected unsatisfactory preparation, especially with respect to the mean scrubbing time, which was lower than the time advocated in the literature [13]. In another study. Nail scrubbing was observed in 33.7 and 41.5% for 2% chlorhexidine and 10% PVPI solutions, respectively. Most healthcare professionals followed all the scrub steps during surgical hand preparation, which promoted an abrasive effect of scrub brush on their skin. This is the reason why the WHO does not recommend the use of scrub brushes [19, 20].

A study involving 156 healthcare professionals in southern Taiwan evaluated the microbial load reduction after surgical hand preparation and found that ABHR had a highly persistent effect (*P* = 0.001) [12]. Another study verified an improved microbial load reduction when an ABHR was employed (1.91–1.52 log10), as compared to chlorhexidine (0.82–1.16 log10) and PVPI (0.52–0.92) solutions [21].

A systematic review of 14 randomized clinical trials compared the efficacy of chlorhexidine, PVPI, and ABHRs. ABHR was as effective as or more effective than antiseptic agent solutions. Nail scrubbing was the surgical hand asepsis step with the lowest evidence level. Moreover, the SSI rates were similar in all cases [17].

In a randomized clinical trial conducted in France between January 01 of 2000 and March 01 of 2001 with 4387 surgical patients, the SSI rates were similar for antiseptic agent solution and ABHR [55 cases (2.4%) vs 53 cases (2.48%)], respectively [18].

Our study presents some important limitations. First, a small imbalance was observed in some of the clinical and demographical features of the patients included in the pre- and in the intervention phases. However, none of those characteristics could affect the primary study outcome, and none of them was actually demonstrated to interfere with the secondary study outcome, in multivariate analysis. Second, compliance with post-discharge follow-up was greater in the pre-intervention than in the intervention period, which could lead to an underestimation of the SSI rate in the intervention period. Third, as the intervention was multifaceted, we are not able to infer the individual impact of each one of its components over the study outcomes.

## Conclusion

The present study provides support for the routine use of ABHR for surgical hand preparation. Our results demonstrate that, when compared to the traditional surgical scrubbing with antimicrobial soap, alcohol-based surgical preparation, along with proper training, may improve the quality of the preparation, reduce the time spent on the preparation, and it is at least equally effective for the prevention of surgical site infections.

## Abbreviations

95% CI: 95% Confidence Interval
ABHR: Alcohol-Based Handrub
Adjusted RR: Adjusted Relative Risk
ASA: American Society of Anesthesiologists physical status classification system
BMI: Body Mass Index
PVPI: Povidone-Iodine
SSI: Surgical Site Infection
WHO: World Health Organization
